# Development of a Malaysian potentially inappropriate prescribing screening tool in older adults (MALPIP): a Delphi study

**DOI:** 10.1186/s40545-023-00630-4

**Published:** 2023-10-19

**Authors:** Chee Tao Chang, Huan Keat Chan, Wee Kooi Cheah, Maw Pin Tan, Alan Swee Hock Ch’ng, Chiann Ni Thiam, Nor Azlina Abu Bakar, Weng Keong Yau, Muhammad Radzi Abu Hassan, Philip Rajan, Kar Choon Tan, Subashini Ambigapathy, Paranthaman Vengadasalam, Surina Zaman Huri, HS Arvinder-Singh, Chern Choong Thum, Wai Mun Chung, Jun How Ooi, Noor Hamizah Sabki, Hooi Peng Lee, Siti Mallissa Mohd Shariff, Muhammad Azuan Azman, Siew Li Teoh, Shaun Wen Huey Lee

**Affiliations:** 1 School of Pharmacy, Monash University Malaysia, Subang Jaya, Malaysia; 2 Clinical Research Centre, Hospital Raja Permaisuri Bainun, Ministry of Health Malaysia, Ipoh, Malaysia; 3 Clinical Research Centre, Hospital Sultanah Bahiyah, Ministry of Health Malaysia, Alor Setar, Malaysia; 4 Department of Medicine, Hospital Taiping, Ministry of Health Malaysia, Taiping, Malaysia; 5 Ageing and Age-Associated Disorders Research Group, Department of Medicine, Faculty of Medicine, Universiti Malaya, Kuala Lumpur, Malaysia; 6 Clinical Research Centre, Hospital Seberang Jaya, Ministry of Health Malaysia, Seberang Jaya, Malaysia; 7 Department of Medicine, Hospital Sultanah Bahiyah, Ministry of Health Malaysia, Alor Setar, Malaysia; 8 Medical Department, Hospital Raja Permaisuri Bainun, Ministry of Health Malaysia, Ipoh, Malaysia; 9 Medical Department, Hospital Kuala Lumpur, Ministry of Health Malaysia, Kuala Lumpur, Malaysia; 10 Hepatological Department, Hospital Selayang, Ministry of Health Malaysia, Selayang, Malaysia; 11 Klinik Kesihatan Buntong, Ministry of Health Malaysia, Ipoh, Malaysia; 12 Klinik Kesihatan Greentown, Ministry of Health Malaysia, Ipoh, Malaysia; 13 Psychiatry Department, Hospital Sultan Ismail, Ministry of Health Malaysia, Johor Bahru, Malaysia; 14 Department of Community Medicine, Hospital University Kebangsaan Malaysia, Bangi, Malaysia; 15 Department of Psychiatry, Hospital Sultan Abdul Aziz Shah, Universiti Putra Malaysia, Serdang, Malaysia; 16 Emergency and Trauma Department, Hospital Taiping, Taiping, Malaysia; 17 Department of Family Medicine, School of Medical Sciences, Universiti Sains Malaysia, Kubang Kerian, Malaysia; 18 Pharmacy Department, Hospital Taiping, Ministry of Health Malaysia, Taiping, Malaysia; 19 Pharmacy Department, Hospital Kuala Lumpur, Ministry of Health Malaysia, Kuala Lumpur, Malaysia; 20 Pharmacy Department, Hospital Sungai Buloh, Ministry of Health Malaysia, Sungai Buloh, Malaysia; 21 Pharmacy Department, Hospital Raja Permaisuri Bainun, Ministry of Health Malaysia, Ipoh, Malaysia; 22 Clinical Research Centre, Hospital Taiping, Ministry of Health Malaysia, Taiping, Malaysia; 23 Department of Medical Sciences, School of Healthcare and Medical Sciences, Sunway University, Petaling Jaya, Malaysia

**Keywords:** Polypharmacy, Potentially inappropriate medications, Older adults, Medication review, Prescribing omission

## Abstract

**Introduction:**

Polypharmacy and potentially inappropriate medications (PIM) are common among older adults. To guide appropriate prescribing, healthcare professionals often rely on explicit criteria to identify and deprescribe inappropriate medications, or to start medications due to prescribing omission. However, most explicit PIM criteria were developed with inadequate guidance from quality metrics or integrating real-world data, which are rich and valuable data source.

**Aim:**

To develop a list of medications to facilitate appropriate prescribing among older adults.

**Methods:**

A preliminary list of PIM and potential prescribing omission (PPO) were generated from systematic review, supplemented with local pharmacovigilance data of adverse reaction incidents among older people. Twenty-one experts from nine specialties participated in two Delphi to determine the list of PIM and PPO in February and March 2023. Items that did not reach consensus after the second Delphi round were adjudicated by six geriatricians.

**Results:**

The preliminary list included 406 potential candidates, categorised into three sections: PIM independent of diseases, disease dependent PIM and omitted drugs that could be restarted. At the end of Delphi, 92 items were decided as PIM, including medication classes, such as antacids, laxatives, antithrombotics, antihypertensives, hormones, analgesics, antipsychotics, antidepressants, and antihistamines. Forty-two disease-specific PIM criteria were included, covering circulatory system, nervous system, gastrointestinal system, genitourinary system, and respiratory system. Consensus to start potentially omitted treatment was achieved in 35 statements across nine domains.

**Conclusions:**

The newly developed PIM criteria can serve as a useful tool to guide clinicians and pharmacists in identifying PIMs and PPOs during medication review and facilitating informed decision-making for appropriate prescribing.

**Supplementary Information:**

The online version contains supplementary material available at 10.1186/s40545-023-00630-4.

## Introduction

The global population is rapidly aging, and it is predicted that the number of adults aged 60 years and above will double from the current 12–22% by 2050 [1]. Older adults are often characterised by multiple comorbidities, which often necessitates treatment with at least five medications, a condition known as polypharmacy [2, 3]. Globally, approximately 45% of the older population is affected by polypharmacy [4], raising concerns about the risk of having potentially inappropriate prescribing (PIP). PIP occurs when the prescribing or potential prescribing omissions (PPO) of medications may result in significant harm for an individual. As such, the challenge is for a prescriber to strike a balance between optimising chronic disease control while minimising the risk of PIP.

PIP which engenders potentially inappropriate medications (PIM) can be addressed by examining the process of prescribing. For instance, studies to date have suggested medication reviews, providing refreshers on pharmacology to prescribers, and clinical decision support systems to reduce the risk of PIM [5, 6]. Another common intervention is deprescribing, or the withdrawal of unnecessary medications under the supervision of a health care professional [7]. To facilitate deprescribing, several screening tools have been developed, including goal-orientated instruments, implicit (judgement-based) criteria tool and explicit (criterion-based) criteria tools. While each type of tool offers their own advantage, the explicit criteria tools are most often adopted in clinical practice due to their simplicity in administration [8]. Examples of explicit criteria tools include the Beers Criteria for Potentially Inappropriate Medication Use in Older Adults (Beers Criteria) [9] as well as the Screening Tool of Older Persons’ Potentially Inappropriate Prescriptions (STOPP), and Screening Tool to Alert Doctors to Right Treatment (START) criteria [10].

The use of these explicit criteria can improve health outcomes among older adults [11]. For example, the implementation of the STOPP–START criteria was shown to reduce falls, delirium episodes, hospital stays, primary and emergency care visits, and medical expenses [12]. Similarly, the use of the EURO–FORTA criteria was shown to reduce adverse drug reactions, minimize inappropriate prescribing of medications, and improve daily living functionality [8, 13]. Nevertheless, due to the differences in therapeutic guidelines and approved medications, several countries have also developed their own explicit criteria, such as those from Taiwan [14], Indonesia [15] and Korea [16, 17]. For example, scopolamine and desiccated thyroid are listed in the STOPP and Beer’s criteria but are unavailable in some markets [9, 10]. The prescribing tools mentioned above were developed in high-income countries using formularies from their sources countries. Lower- and middle-income countries, such as Malaysia, and other low resource settings, however, often have a more limited formulary. Therefore, the development of a list specifically for the LMIC and low resource context may be important given the differences in formularies and prescribing practices.

Our systematic review also found that most explicit PIM criteria often did not meet all the necessary quality components [18]. Importantly, most of these criteria were formulated using pre-established frameworks, without taking the latest pharmacovigilance data and current literature into account, which offers a rich and valuable source of data often not detected in previous studies [18].

## Research question

What are the medications or medication classes that can be identified as potentially inappropriate and potential prescribing omission for older adults living in Malaysia, considering both disease-independent and disease-dependent factors, and how do they relate to the existing criteria, pharmacovigilance data and real-world medication profiles of older adults?

## Aim of the study

This study aims to develop a tailored list to facilitate deprescribing of potentially inappropriate medications and identifications of omitted drugs (MALPIP) for the older population through systematic review of established PIM criteria, pharmacovigilance data and Delphi interviews.

## Ethics approval

This study was registered in the National Medical Research Registry (NMRR-22-02443-CAL) and approved by Malaysian Research Ethics Committee (22-024423-CAL) on the 7th December 2022.

## Methods

The MALPIP list was developed in four phases. To ensure the comprehensiveness and relevance of the list, a systematic review of Asian studies and Western PIM criteria (Phase 1) was performed, followed by review of pharmacovigilance data (Phase 2) to develop the preliminary PIP lists (Phase 3). In Phase 4, the final MALPIP criteria was generated through Delphi rounds and interviews with the panellists.

### Phase 1: systematic review of Asian PIM and PPO criteria supplemented by four Western PIM criteria

A systematic literature study was conducted on seven databases (Medline, EMBASE, CINAHL, PsyInfo, PubMed, Web of Science and Cochrane Library) to identify studies that reported the explicit PIM and PPO lists for older adults that were published involving Asian populations [18].

The lists of PIM and PPO from each of the identified studies were complied, along with the scientific evidence and their potential harm. The lists of PIM and PPO were also separated based on medication class and disease. This was supplemented with four of the most commonly used Western PIP criteria, namely, BEERS [9], STOPP–START [10], PRISCUS criteria [19] and French criteria [20].

### Phase 2: review of pharmacovigilance data

Pharmacovigilance data from the Malaysian National Pharmaceutical Regulatory Agency (NPRA) in 2020 and 2021 were reviewed, which included 874 ingredients and 22,035 adverse reaction incidents among people over the age of 60. Duplicates were removed, and the following medication classes were excluded: antibiotics, chemotherapy drugs, tuberculosis medications, antiviral, antifungal, antivenin, toxoid vaccines, anaesthetic agent, and contrast agent.

The remaining medications were further sorted based on the event: hospitalization, life-threatening event or death. Any medication or medication classes which had reported at least one event of hospitalization, life-threatening event or death were extracted.

### Phase 3: development of a preliminary list of PIPs

The list of drugs identified from Phase 1 and 2 were collated. Medications that were not registered in the Malaysian product registration and licensing system were excluded (Quest 3 + web portal, NPRA, Ministry of Health, Malaysia). Drugs that fall under the same category were merged into pharmacological classes.

The drugs were categorized into three lists: independent PIM, disease-specific PIM, and PPO. The lists included information on each medication and their reasons being listed as a PIM or PPO, together with the supporting evidence. The three preliminary lists were then reviewed by two authors to ensure consistency. The preliminary list was transformed into a web-based questionnaire and distributed via Google Form.

### Phase 4: generation of MALPIP through Delphi and interviews

The Delphi method was employed, since it allowed for a structured approach to leverage the collective opinions of a group of experts and subsequently address subject matter that did not have conclusive evidence through a series of surveys [21]. The report of the findings was guided by nine Delphi quality criteria outlined by Nasa and colleague [21].

### Selection and anonymity of panel

Experts comprised of healthcare professional who had extensive knowledge on diseases or medications used among older population, and had more than 5 years of experience in providing integrated care to older adults. Experts were recruited through the personal network of the researchers and snowballing.

Twenty-one experts from nine specialties, including geriatric medicine (*n* = 6), general practice (*n* = 1), psychiatry (*n* = 2), internal medicine (*n* = 2), otorhinolaryngology (*n* = 1), geriatric pharmacy (*n* = 4), family medicine (*n* = 3), clinical research (*n* = 1) and emergency (*n* = 1) participated in the study (Table 1). Anonymity was secured in both Delphi rounds to prevent dominance and group conformity [21].

**Table 1:** Demographic information of participants in two Delphi rounds (*n* = 21)

Characteristics	Frequency (%)
Gender	
Male	13 (61.9%)
Female	8 (38.1%)
Age, years (median, IQR)	41.0 (33.4–48.0)
Working experience, years (median, IQR)	16.0 (8.6–23.5)
Specialties	
Geriatric medicine	6
Geriatric pharmacy	4
Family medicine	3
Internal medicine	2
Psychiatry	2
General practice	1
Otorhinolaryngology	1
Emergency medicine	1
Clinical research	1
Work settings	
Tertiary hospital	14
Primary health clinic	3
University hospital	2
Ministry of Health	2

### Iterative Delphi rounds

The first Delphi round was conducted in February 2023. The web-based questionnaire was distributed through email, and the respondents completed the survey at a time of convenient to them. Panellists were given 2 weeks to respond, and reminders were sent to non-responders at day 7 and day 10.

The independent PIM list were rated based on a five-point Likert scale, ranging from either “definitely PIM, potential PIM, undecided, not PIM to definitely not PIM”; and PPO rated as “definitely should be started, should be started, undecided, should not be started, definitely should not be started”. For disease-specific PIM, the experts were asked whether they “agree” or “disagree” to the list of PIM specific to each disease. Experts were also asked to comment and propose amendments (e.g., addition or removal of item). The feedback from the first round were compiled and analyzed.

### Controlled feedback

Using a controlled feedback method [21], anonymized responses from the first round were analyzed, summarized and shared with the panellists. Panel members were allowed to view their own response and responses from other members and were allowed to alter their decision in the second round. In addition, a summary of the PIM and PPO that had not achieved consensus in the first round were generated and shared. This included the rationale of the item listed as a PIM or PPO candidates, and the relevant references in an Excel file.

The second Delphi round was conducted in March 2023 and involved the same group of panellists. A questionnaire that only covered the item for which consensus was not achieved, along with additional items that were proposed during the first round were sent to the panellists. For medications that did not reach consensus after two rounds of Delphi, one-to-one interview with six geriatricians were performed to reach a final decision.

### Definition of consensus, analysis of consensus and closing criteria

For independent PIM and PPO, consensus was reached if the upper limit of the 95% confidence interval (CI) was less than 3.0 (PIM/PPO) or if the lower bound of the CI was more than 3.0 (not PIM/PPO). If the upper and lower 95% CI limit of an item falls on both sides of 3.0, consensus was not achieved, and the item were asked again in the second Delphi round.

For disease-specific PIM, consensus was reached if ≥ 80% (17 of 21 members) agreed with the medications listed for each specific disease. Item that did not achieve consensus in the first round were asked in the second round. When consensus could not be achieved after two Delphi rounds, decision was made through a majority voting (> 50%) with six geriatricians.

### Data analysis and stability

Data were analyzed using SPSS version 27 (SPSS Inc., Chicago, IL, USA). Results were presented as mean with corresponding 95% confidence interval for PIM independent of disease and PPO. Disease-specific PIM were summarized descriptively. The stability of the results in the two Delphi rounds were tested using Kendall's *W* coefficient of concordance, with values ranging from 0 to 1. Higher values indicate stronger correlation between expert on the proposed criteria. In addition, a coefficient of variation (CV) was used to measure the variance of expert opinions, with lower CV value of 0.25 deemed as acceptable [22].

To aid interpretation, we also compared the overlap of medication classes between MALPIP with the four most commonly used PIP criteria, namely, STOPP–START, Beers, PRISCUS and French criteria. We assumed that if a medication was identified as inappropriate by most of the listed criteria, it indicates the universal needs to deprescribe the particular medication in different patient populations rather than if it was only identified by one set of criteria.

To assess the validity of the final MALPIP list, we examined the applicability of the generated criteria in a data set of 553 older adults aged 60 years and above who were hospitalized with COVID-19 from five Malaysian public hospitals. We examined the proportion of patients who received at least one type of PIM, total number of PIM and types of PIM, and compared them to the STOPP and Beers criteria [23].

## Results

### Development of preliminary PIP list

Our systematic review [18] identified a total of 311 PIM, 42 disease-specific criteria PIM and 34 PPO candidates (Phase 1). Two additional drug classes (Dipeptidyl peptidase-4 inhibitor (DPP4)-inhibitors and statin) and 17 individual medications were also identified as potentially inappropriate, bringing a total of 406 items (330 PIM, 42 disease-specific criteria and 34 PPO candidates) in the draft MALPIP list.

**Fig. 1:**
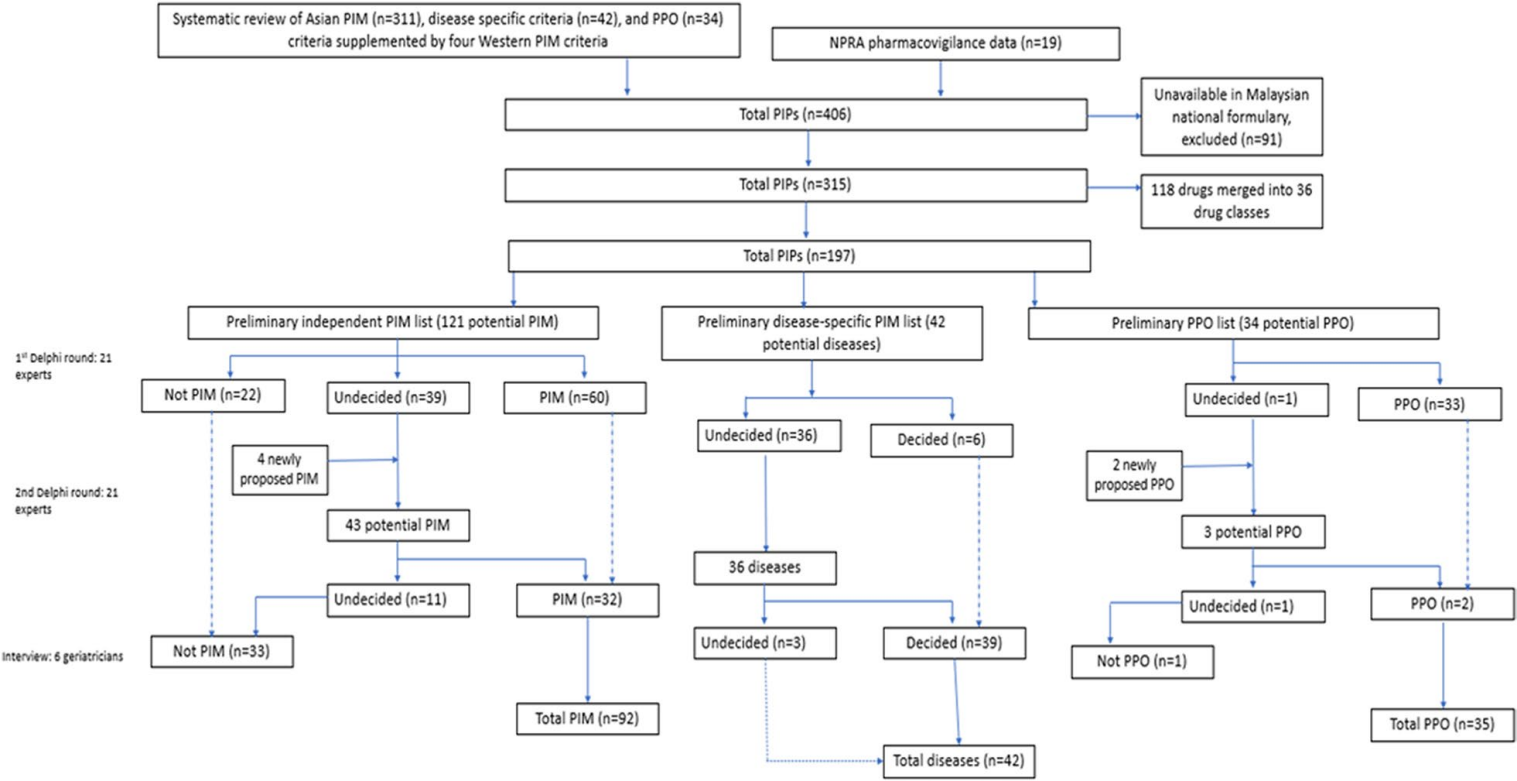
Delphi process to identify independent PIM, disease-specific PIM and PPO

Initial review of the draft list excluded 91 medications, since these were unavailable in the Malaysian market. We also merged 118 unique medications into 36 drug classes (e.g., H2-receptor antagonists consisted of cimetidine and famotidine, Additional file 1: Table S1). The final MALPIP draft included 121 independent PIM, 42 disease-specific criteria PIM and 34 PPO candidates which were included in the Delphi (Fig. 1).

#### Independent potentially inappropriate medications (PIM)

In the first Delphi round, 60 medications (25 drug classes, 35 unique drugs; 49.6%) were unequivocally decided as PIM, 39 medications (32.2%) remained undecided and 22 medications (3 drug class, 19 unique drugs; 18.2%) were decided as definitely not PIM. Experts also proposed an additional four medications, (lithium, sodium valproate, levetiracetam and transdermal fentanyl) that were not identified in our initial review as a PIM (Table 2).

**Table 2:** PIM list independent of disease

PIM criteria	Mean score (Round 1)	95% Confidence Interval (Round 1)	Mean score (Round 2)	95% Confidence Interval (Round 2)	Decision
*Acid related disorders (A02)*					
Aluminium antacids	3.48	2.96–3.99	2.62	2.23–3.01	Not PIM^a^
H2 receptors antagonists	2.76	2.21–3.32	2.19	1.88–2.50	PIM
Proton pump inhibitors	2.62	2.00–3.24	2.19	1.74–2.63	PIM
Metoclopramide	2.33	1.83–2.84	–	–	PIM
Atropine	1.52	1.29–1.76	–	–	PIM
Belladona alkaloids	1.71	1.38–2.04	–	–	PIM
Clidinium–chlordiazepoxide	1.86	1.53–2.19	–	–	PIM
Dicyclomine	2.00	1.71–2.29	–	–	PIM
Homatropine	1.95	1.68–2.22	–	–	PIM
*Constipation (A06)*					
Viscous paraffin	3.10	2.49–3.70	2.48	1.99–2.97	PIM
Bisacodyl	3.10	2.47–3.72	2.67	2.12–3.21	Not PIM^a^
Cascara sagrada	2.95	2.62–3.29	2.47	2.11–2.85	PIM
Magnesium oxide	3.14	2.75–3.53	2.52	2.13–2.92	PIM^b^
Polyethylene glycol	3.05	2.52–3.58	2.48	2.03–2.92	PIM^b^
*Diabetes (A10)*					
Metformin	3.48	3.01–3.97	–	–	Not PIM
Sulfonylureas (long acting)	2.14	1.60–2.69	–	–	PIM
Pioglitazone	2.14	1.73–2.56	–	–	PIM^b^
Insulin sliding scale	2.81	2.24–3.38	2.38	1.83–2.93	PIM
Acarbose	3.57	3.12–4.01	–	–	Not PIM
Sodium–glucose co-transporters (SGLT) inhibitors	3.29	2.82–3.74	2.81	2.34–3.28	Not PIM^a^
DPP4 inhibitors	4.33	3.79–4.88	–	–	Not PIM
*Antithrombotic agents (B01)*					
Aspirin	2.86	2.37–3.34	2.81	2.30–3.32	Not PIM^a^
Clopidogrel	2.95	2.42–3.48	2.67	2.18–3.15	Not PIM^a^
Dipyridamole	2.52	2.01–3.04	2.24	1.86–2.62	PIM
Vitamin K antagonists	2.43	2.00–2.85	–	–	PIM^b^
Direct thrombin inhibitors	2.71	2.28–3.15	2.48	2.01–2.95	PIM
Factor Xa inhibitors	2.71	2.30–3.13	2.33	1.87–2.80	PIM
Ticlopidine	2.43	1.98–2.87	–	–	PIM
Prasugrel	2.33	1.89–2.77	–	–	PIM
Enoxaparin	3.83	2.80–4.87	–	–	Not PIM
Heparin	3.83	3.04–4.62	–	–	Not PIM
Fondaparinux	4.00	3.34–4.66	–	–	Not PIM
Ticagrelor	4.00	3.34–4.66	–	–	Not PIM
Streptokinase	3.83	3.04–4.62	–	–	Not PIM
*Anaemic preparations (B03)*					
Oral iron	3.24	2.68–3.79	3.05	2.56–3.54	Not PIM^a^
*Cardiac therapy (C01)*					
Digoxin	2.05	1.65–2.44	–	–	PIM
Antiarrhythmics	2.19	1.79–2.59	–	–	PIM
Ivabradine	4.17	3.74–4.59	–	–	Not PIM
Isosorbide dinitrate	4.00	3.06–4.93	–	–	Not PIM
Isosorbide mononitrate	3.83	3.04–4.62	–	–	Not PIM
*Antihypertensives (C02)*					
Methyldopa	1.90	1.52–2.28	–	–	PIM
Clonidine	2.00	1.59–2.40	–	–	PIM
Moxonidine	2.52	2.07–2.97	–	–	PIM
Doxazosin	2.29	1.78–2.79	–	–	PIM
Prazosin	2.29	1.78–2.79	–	–	PIM
Reserpine	1.90	1.56–2.25	–	–	PIM
*Diuretics (C03)*					
Loop diuretics	2.33	1.83–2.84	–	–	PIM
Thiazide diuretics	2.29	1.83–2.74	–	–	PIM
Spironolactone	2.38	1.92–2.85	–	–	PIM
*Peripheral vasodilators (C04)*					
Pentoxifylline	2.81	2.26–3.36	2.62	2.13–3.11	Not PIM^a^
*Beta blocking agents (C07)*					
Non-selective beta-blocker	2.33	1.94–2.72	–	–	PIM
*Calcium channel blockers (C08)*					
Non-dihydropyridine calcium channel blocker	2.43	1.98–2.87	–	–	PIM^b^
Dihydropyridine calcium channel blocker	2.43	1.91–2.94	–	–	PIM
Felodipine	4.00	3.06–4.93	–	–	Not PIM
*Agents acting on the renin–angiotensin system (C09)*					
ACE inhibitors	3.48	2.95–4.00	3.10	2.60–3.60	Not PIM^a^
ARBs	3.57	3.06–4.08	–	–	Not PIM
Sacubitril/valsartan combination	3.83	3.04–4.62	–	–	Not PIM
*Lipid modifying agents (C10)*					
Statin	4.00	3.34–4.67	–	–	Not PIM
Gemfibrozil	4.00	3.34–4.67	–	–	Not PIM
Fenofibrate	4.17	3.74–4.59	–	–	Not PIM
*Sex hormones and modulators of genital systems (G03)*					
Oral oestrogens	2.67	2.18–3.15	2.33	1.89–2.77	PIM
Androgens	2.48	2.05–2.90	–	–	PIM
Megestrol	2.29	1.85–2.72	–	–	PIM
*Urological (G04)*					
Phosphodiesterase-5 inhibitors	2.38	1.83–2.92	–	–	PIM^b^
Antimuscarinics for urinary frequency and incontinence	2.19	1.72–2.66	–	–	PIM
Selective alpha-1 blockers	2.76	2.23–3.30	2.33	1.87–2.80	PIM
*PITUITARY AND HYPOTHALAMIC HORMONES AND ANALOGUES (H01)*					
Desmopressin	2.43	1.94–2.92	–	–	PIM
*Corticosteroids for systemic use (H02)*					
Systemic corticosteroids	2.19	1.79–2.59	–	–	PIM
*Hormonal preparations (H03)*					
Levothyroxine	3.24	2.76–3.71	2.86	2.40–3.32	Not PIM^a^
Carbimazole	4.17	3.74–4.59	–	–	Not PIM
*Antibacterial for systemic use (J01)*					
Nitrofurantoin	2.71	2.21–3.22	2.48	2.05–2.90	PIM
Vancomycin	2.81	2.36–3.26	2.43	1.92–2.94	PIM^b^
Clindamycin	2.86	2.40–3.32	2.52	2.13–2.92	PIM^b^
Aminoglycosides	2.57	2.15–2.99	–	–	PIM^b^
*Antineoplastic agents (L01)*					
Growth hormone	2.43	2.09–2.77	–	–	PIM
Monoclonal antibodies	3.00	2.62–3.38	2.43	2.01–2.85	PIM
*Immunosuppressants (L04*)					
Leflunomide	2.90	2.45–3.36	2.24	1.81–2.67	PIM^b^
Methotrexate	2.71	2.26–3.17	2.10	1.67–2.52	PIM^b^
Azathioprine	2.81	2.34–3.29	2.24	1.79–2.69	PIM^b^
Etanercept	2.90	2.46–3.36	2.24	1.81–2.67	PIM^b^
*Anti-inflammatory and antirheumatic products (M01)*					
Non-COX-2 selective NSAIDs	1.81	1.39–2.23	–	–	PIM
COX-2 selective inhibitors	2.24	1.83–2.64	–	–	PIM
*Muscle relaxants (M03)*					
Baclofen	2.00	1.57–2.43	–	–	PIM
Orphenadrine	1.81	1.50–2.11	–	–	PIM
Chlorzoxazone	2.10	1.72–2.47	–	–	PIM
*Antigout preparations (M04)*					
Colchicine	2.90	2.37–3.44	2.48	2.03–2.92	PIM
*Drugs for treatment of bone diseases (M05)*					
Oral bisphosphonates	3.38	2.99–3.77	2.81	2.34–3.28	Not PIM^a^
*Analgesics (N02)*					
Opioids	2.19	1.82–2.56	–	–	PIM
Ergotamine and derivatives	2.24	1.81–2.67	–	–	PIM
Paracetamol	4.29	4.03–4.54	–	–	Not PIM
Fentanyl patch	–	–	2.38	1.96–2.80	PIM^a^
*Antiepileptics drugs (N03)*					
Barbiturates	1.86	1.50–2.22	–	–	PIM
Phenytoin	2.10	1.67–2.52	–	–	PIM^b^
Lithium	–	–	1.95	1.58–2.32	PIM^b^
Levetiracetam	–	–	3.14	2.60–3.69	Not PIM^a^
Sodium valproate	–	–	2.33	1.89–2.77	PIM^a,b^
Carbamazepine	3.83	3.04–4.62	–	–	Not PIM
*Antiparkinson agents (N04)*					
Trihexyphenidyl (benzhexol)	2.00	1.62–2.38	–	–	PIM
Biperiden	2.24	1.89–2.59	–	–	PIM^b^
Benzatropine	2.10	1.74–2.45	–	–	PIM
Selegiline	2.62	2.13–3.11	2.48	2.11–2.85	PIM^b^
Levodopa and dopamine agonists	3.10	2.62–3.57	2.48	2.03–2.92	PIM^b^
*Neuroleptics (N05)*					
Barbiturates with hypnotic properties	1.81	1.47–2.15	–	–	PIM
Atypical antipsychotics	1.95	1.62–2.29	–	–	PIM
Thioxanthones	2.19	1.85–2.53	–	–	PIM^b^
Chloral hydrate	2.19	1.79–2.59	–	–	PIM
Benzodiazepines	1.67	1.40–1.92	–	–	PIM
Phenothiazines (first generation antipsychotics)	1.86	1.64–2.07	–	–	PIM
Haloperidol	1.90	1.59–2.22	–	–	PIM
Zopiclone	2.05	1.65–2.44	–	–	PIM
Zolpidem	2.10	1.67–2.52	–	–	PIM
Hydroxyzine	1.76	1.56–1.96	–	–	PIM
*Neuroanaleptics (N06)*					
Selective serotonin re-uptake inhibitors (SSRIs)	2.62	2.15–3.09	2.38	1.99–2.77	PIM
Acetylcholinesterase inhibitors	2.86	2.33–3.38	2.48	2.05–2.90	PIM^b^
Tricyclic antidepressants	1.62	1.39–1.84	–	–	PIM
Piracetam	2.90	2.43–3.38	2.14	1.78–2.50	PIM
Methylphenidate	2.33	1.92–2.75	–	–	PIM^b^
Mirtazapine	3.83	3.04–4.62	–	–	Not PIM
Venlafaxine	3.83	3.04–4.62	–	–	Not PIM
*Antivertigo (N07)*					
Flunarizine	2.76	2.24–3.28	2.29	1.93–2.64	PIM^b^
Cinnarizine	2.71	2.21–3.22	2.43	2.03–2.82	PIM^b^
*Antimalarials (P01)*					
Hydroxychloroquine	2.17	1.38–2.96	–	–	PIM^b^
*Drugs for obstructive airway diseases (R03)*					
Xanthine derivatives	2.24	1.81–2.67	–	–	PIM
Anti-muscarinic bronchodilators	3.29	2.73–3.85	2.48	2.05–2.90	PIM^b^
*Antihistamines for systemic use (R06)*					
First generation antihistamines	1.75	1.47–2.25	–	–	PIM

*TCA* tricyclic antidepressants, *SSRI* selective serotonin reuptake inhibitors, *NSAIDs* nonsteroidal anti-inflammatory drug, *ACE* angiotensin-converting-enzyme, *ARB* angiotensin receptor blockers

^a^Decision made in 3rd round interview with 6 geriatricians by majority votes

^b^Not designated as independent PIM in STOPP, Beers, PRISCUS and French criteria

In the second Delphi round, 43 independent PIM candidates were assessed. Experts identified another 32 medications (12 drug classes, 20 unique drugs; 26.4%) that could be included as PIM. Eleven medications (4 drug classes, 7 unique drugs; 9.1%) could not be unambiguously classified even after the second round of Delphi. Interview with experts comprising of geriatricians voted in consensus that these 11 medications should be excluded from the PIM list. The complete list of independent PIM and practice statements are available in Additional file 1: Table S2.

#### Disease-specific PIM

In the disease-specific PIM list, expert panel reached consensus in 6 out of 42 (14.3%) disease-specific PIM criteria and provided suggestions for the remaining 36 criteria (85.7%). In the second Delphi, consensus was reached for 33 out of 36 criteria (91.7%) (Table 3). Interview with the experts suggested that only one of the criteria was PIM with the remaining two as not a PIM. The complete list of disease-dependent PIM and practice statements are available in Additional file 1: Table S3.

**Table 3:** Disease-specific PIM lists

Condition	Medications
*Circulatory system*	
Heart failure	Non-COX-2 selective NSAIDs, COX-2 selective NSAIDs, thiazolidinediones, non-dihydropyridine calcium channel blockers (diltiazem, verapamil), digoxin, tricyclic antidepressants, sildenafil, tadalafil, vardenafil
Syncope	Donepezil, rivastigmine, galantamine, non-selective alpha-blockers, tricyclic antidepressants, benzodiazepines, antipsychotics, methyldopa, venlafaxine
Bradycardia	Beta-blockers, donepezil, rivastigmine, galantamine, non-dihydropyridine calcium channel blockers
Arrythmias	Antiarrhythmics, tricyclic antidepressants, antipsychotics, donepezil, quinine
Hypertension	Loop diuretics, non-selective alpha blockers, NSAIDs, thiazides, methyldopa
Hypertension and urinary incontinence	Loop diuretics
Dependent ankle oedema	Loop diuretics, calcium channel blockers
Hypokalemia	Thiazide, digoxin, fludrocortisone, loop diuretics,
Hyponatremia	Thiazides, selective serotonin reuptake inhibitors
Hypercalcemia	Thiazides
Hyperkalemia	ACE inhibitors, ARBs, spironolactone
High bleeding risk (e.g., coagulopathy)	Antiplatelet, vitamin K antagonists, direct thrombin inhibitors, direct factor Xa inhibitors, NSAIDs
First deep vein thrombosis (without continuing risks)	Vitamin K antagonists, direct thrombin inhibitors, direct factor Xa inhibitors
Cardiac conduction abnormalities and heart block	Tricyclic antidepressants, digoxin, verapamil, amiodarone, acetylcholinesterase inhibitors (e.g., donepezil, rivastigmine), beta blockers
Orthostatic hypotension	Non-selective alpha blockers, calcium channel blockers, long-acting nitrates, chlorpromazine, tricyclic antidepressants, hydralazine, thiazide, loop diuretics, dopamine agonists (except levodopa), selegiline, antipsychotics, SGLT-2 inhibitors
Acute coronary symptoms	COX-2 selective NSAIDs, Non-COX-2 selective NSAIDS
Raynaud disease	Beta-blockers
*Nervous system*	
Delirium, dementia or cognitive impairment	Urological spasmolytic (e.g., oxybutynin, tolterodine), first-generation antihistamines, antiparkinsonian drugs, antiemetics, muscle relaxants (e.g., orphenadrine), tricyclic antidepressants, paroxetine, antipsychotics, disopyramide, intestinal antispasmodics (e.g., atropine, scopolamine), psychostimulants (methylphenidate), barbiturates, benzodiazepines, zolpidem, deferoxamine, testosterone, corticosteroids, dopamine agonist, antiepileptics
History of falls or fractures	Antipsychotics, benzodiazepines, zolpidem, opioids, tricyclic antidepressants, antiepileptics (e.g., carbamazepine, phenytoin), first generation antihistamines, non-selective alpha blockers, non-selective beta-blockers, paroxetine, duloxetine, loop diuretics
Parkinson disease and Parkinsonism	Antipsychotics (except quetiapine, clozapine), antiemetics (e.g., prochlorperazine, promethazine, metoclopramide), benzhexol
Behavioral and psychological symptoms in dementia	Antipsychotics, anticonvulsants, benzodiazepines
Sleep disorders or insomnia	Antipsychotics, psychostimulants (e.g., methylphenidate, caffeine), theophylline, pseudoephedrine, benzodiazepines (temazepam could be considered for short-term use), barbiturates, tricyclic antidepressants, Z-hypnotics
Benign essential tremor	Levodopa and dopamine agonists, benzhexol
Depression	Centrally acting antihypertensives (e.g., methyldopa, reserpine), tricyclic antidepressant
Seizures or epilepsy	Antipsychotics, bupropion, antidepressants, beta-lactam antibiotics, theophylline, H2 antagonists, levodopa, isoniazid, opioids
*Gastrointestinal system*	
History of gastric or duodenal ulcers	Aspirin, Non-COX-2 Selective NSAIDs, Corticosteroids
History of esophageal ulcers and dysphagia	Oral bisphosphonates
Constipation	Drugs with anticholinergics properties, verapamil, oral iron, opioids, aluminium antacids, tricyclic antidepressants, calcium supplements, diuretics
*Genitourinary system*	
Chronic kidney disease	Non-COX-2 selective NSAIDs, COX-2 selective NSAIDs, glibenclamide, digoxin, dabigatran, metformin, oral bisphosphonates, colchicine, thiazide, factor Xa inhibitors, donepezil, memantine, cimetidine, ciprofloxacin, gabapentin, PPI, ACEI, ARBs, warfarin, allopurinol, opioids, aminoglycosides, amphotericin B, vancomycin, acyclovir, clopidogrel, ticlopidine
Benign prostatic hyperplasia	Strong anticholinergic drugs, tricyclic antidepressants, antimuscarinic bronchodilators (e.g., ipratropium, tiotropium)
Urinary incontinence	Non-selective alpha blockers, tricyclic antidepressants, diuretics
*Respiratory system*	
Bronchial asthma	Non-selective beta blockers, benzodiazepines
Chronic obstructive pulmonary disease	Theophylline, non-selective beta-blockers, benzodiazepines
Acute or chronic respiratory failures	Benzodiazepines, opioids, alcohol, cocaine, amphetamines
Sleep apnea	Benzodiazepines
*Other systems*	
Narrow angle glaucoma	Tricyclic antidepressants, antimuscarinic bronchodilators, strongly anticholinergic drugs, Urological spasmolytic (e.g., oxybutynin)
Osteoarthritis and osteoporosis	Systemic corticosteroids (except intra-articular), high dose calcium supplements, NSAIDs, eperisone
Pain	Opioids in chronic mild pain, long-acting opioids without short-acting opioids in breakthrough pain, benzodiazepines, tramadol
Gout	Loop diuretics, colchicine
Diabetes mellitus	Beta blockers, corticosteroids, olanzapine, quetiapine, risperidone
Breast cancer	Oestrogens
Venous thromboembolism	Oestrogens

*NSAIDs* nonsteroidal anti-inflammatory drug

#### Potential drug omission

In the first Delphi round, 33 of the 34 medications (97.1%) were unequivocally agreed as PPO in older adults. Experts also proposed two additional PPO criteria: (i) single dose of herpes zoster vaccines for people 60 years or older, with or without a prior episode of herpes zoster and (ii) Tetanus, diphtheria, and pertussis vaccines (TDaP) to be given to older adults 65 years and older. Experts unequivocally agreed that Zoster and DTaP vaccines should be listed as PPO in older population in the second Delphi, while aspirin use in atrial fibrillation was decided as “not PPO” after the interview (Table 4).

**Table 4:** Omitted treatments that could be restarted

START criteria	Mean scores	95% confidence interval
*Cardiovascular system*		
Vitamin K antagonists or direct thrombin inhibitors or factor Xa inhibitors in the presence of chronic atrial fibrillation	2.10	1.64–2.55
Antiplatelet therapy (aspirin or clopidogrel or prasugrel or ticagrelor) with a documented history of coronary, cerebral or peripheral vascular disease	1.71	1.39–2.04
Antihypertensive therapy where systolic blood pressure consistently > 160 mmHg and/or diastolic blood pressure consistently > 90 mmHg; if systolic blood pressure > 140 mmHg and/or diastolic blood pressure > 90 mmHg, if diabetic	2.24	1.89–2.59
Statin therapy with a documented history of coronary, cerebral or peripheral vascular disease, unless the patient’s status is end-of-life or age is > 85 years	1.90	1.59–2.22
Angiotensin Converting Enzyme (ACE) inhibitor with systolic heart failure and/or documented coronary artery disease	1.67	1.40–1.93
Beta-blocker with ischaemic heart disease	1.95	1.56–2.35
Appropriate beta-blocker (bisoprolol, nebivolol, metoprolol or carvedilol) with stable systolic heart failure	1.71	1.42–2.01
*Respiratory system*		
Regular inhaled b-2agonist or antimuscarinic bronchodilator (e.g., ipratropium, tiotropium) for mild to moderate asthma or COPD	1.76	1.48–2.05
Regular inhaled corticosteroid for moderate–severe asthma or COPD	1.81	1.47–2.15
Home continuous oxygen with documented chronic hypoxaemia	2.10	1.71–2.47
*Central nervous system and eyes*		
L-DOPA or a dopamine agonist in idiopathic Parkinson’s disease with functional impairment and resultant disability	2.00	1.54–2.46
Non-TCA antidepressant drug in the presence of persistent major depressive symptoms	1.86	1.47–2.25
Acetylcholinesterase inhibitor (e.g., donepezil, rivastigmine, galantamine) for mild–moderate Alzheimer’s dementia or Lewy Body dementia	2.05	1.63–2.47
Topical prostaglandin, prostamide or beta-blocker for primary open-angle glaucoma	2.05	1.68–2.41
Selective serotonin reuptake inhibitor (or SNRI or pregabalin if SSRI contraindicated) for persistent severe anxiety that interferes with independent functioning	1.95	1.58–2.32
Dopamine agonist (ropinirole or pramipexole or rotigotine) for Restless Legs Syndrome, once iron deficiency and severe renal failure have been excluded	2.43	2.03–2.82
*Gastrointestinal*		
Proton Pump Inhibitor with severe gastro-oesophageal reflux disease or peptic stricture requiring dilatation	1.62	1.39–1.85
Fibre supplements (e.g., bran, ispaghula, methylcellulose, sterculia) for diverticulosis with a history of constipation	2.10	1.78–2.41
*Musculoskeletal system*		
Disease-modifying anti-rheumatic drug (DMARD) with active, disabling rheumatoid disease	1.95	1.51–2.39
Bisphosphonates and vitamin D and calcium in patients taking long-term systemic corticosteroid therapy	2.33	1.92–2.75
Vitamin D and calcium supplement in patients with known osteoporosis and/or previous fragility fracture(s) and/or (Bone Mineral Density *T*-scores more than − 2.5 in multiple sites)	1.76	1.52–2.01
Bone anti-resorptive or anabolic therapy (e.g., bisphosphonate, teriparatide, denosumab) in patients with documented osteoporosis, where no pharmacological or clinical status contraindication exists (Bone Mineral Density *T*-scores—> 2.5 in multiple sites) and/or previous history of fragility fracture(s)	2.05	1.65–2.44
Vitamin D supplement in older people who are housebound or experiencing falls or with osteopenia (Bone Mineral Density *T*-score is > − 1.0	2.00	1.62–2.38
Xanthine–oxidase inhibitors (e.g., allopurinol, febuxostat) with a history of recurrent episodes of gout	1.95	1.68–2.22
Folic acid supplement in patients taking methotrexate	1.67	1.40–1.93
*Endocrine*		
ACE inhibitor or Angiotensin Receptor Blocker (if intolerant of ACE inhibitor) in diabetes with evidence of renal disease, i.e., dipstick proteinuria or microalbuminuria (> 30 mg/24 h) with or without serum biochemical renal impairment	1.48	1.20–1.75
*Urogenital*		
Alpha-1 receptor blocker with symptomatic prostatism, where prostatectomy is not considered necessary	1.95	1.59–2.32
5-Alpha reductase inhibitor with symptomatic prostatism, where prostatectomy is not considered necessary	2.00	1.68–2.32
Topical vaginal oestrogen or vaginal oestrogen pessary for symptomatic atrophic vaginitis	2.24	1.89–2.59
*Opioids*		
High-potency opioids in moderate–severe pain, where paracetamol, NSAIDs or low-potency opioids are not appropriate to the pain severity or have been ineffective	1.81	1.58–2.04
Laxatives in patients receiving opioids regularly	1.71	1.36–2.07
*Vaccines*		
Seasonal trivalent influenza vaccine annually	1.57	1.30–1.84
Pneumococcal vaccine at least once after age 65 according to national guidelines	1.62	1.31–1.92
Single dose of herpes zoster vaccines for people 60 years or older, with or without a prior episode of herpes zoster (shingles)**	2.10	1.72–2.47^a^
Tetanus, diphtheria, and pertussis vaccines (TDaP) to be given to older adults 65 years and older**	2.14	1.81–2.47^a^
*Drugs that should not be restarted*		
Aspirin (75–160 mg once daily) in the presence of chronic atrial fibrillation, where Vitamin K antagonists or direct thrombin inhibitors or factor Xa inhibitors are contraindicated*	2.90	2.37–3.44^b^

*PPO* potential prescribing omission, *COPD* chronic obstructive pulmonary disease, *TCA* tricyclic antidepressants, *SNRI* serotonin–norepinephrine reuptake inhibitor, *SSRI* selective serotonin reuptake inhibitors, *NSAIDs* nonsteroidal anti-inflammatory drug, *ACE* angiotensin-converting-enzyme, *ARB* angiotensin receptor blockers

^a^Proposed after first round and achieved consensus at second round

^b^Did not achieve consensus at second round (mean: 3.10, CI 2.56–3.63). Decision made based on interviews with 6 geriatricians by majority votes

### Stability

Kendall's *W* coefficient of concordance found that opinions of all the experts are generally in agreement in both Delphi. Similarly, the coefficient of variation in the first (0.226) and second (0.232) Delphi round were ≤ 0.25, indicating satisfactory stability.

### MALPIP criteria

At the end of the expert round, a total of 92 PIM independent of disease, 42 disease-specific PIM and 35 PPO were included in the MALPIP. The finalized MALPIP list is available at https://sites.google.com/moh.gov.my/malpip/home, and the corresponding Android application can be downloaded from Google Play Store at https://play.google.com/store/apps/details?id=pack.geridea&pli=1.

When compared with STOPP–START, Beers, PRISCUS, and French criteria, MALPIP had the highest overlap with STOPP–START criteria (54.7%), followed by Beers criteria (38.5%), PRISCUS (35.9%), and French criteria (21.5%). MALPIP also included 27 medications from 16 medication classes that did not appear in any of the four criteria (Table 1). In addition, we also identified four diseases that did not appear in any of the criteria, i.e., Raynaud disease, depression, seizures and sleep apnoea.

### Application of MALPIP

Using secondary data [23], the MALPIP list identified a total of 374 older adults (67.6%) who had at least one PIM detected, compared to 124 older adults (22.4%) using the Beers criteria or 104 (18.8%) older adults using the STOPP (104/553, 18.8%).

In terms of PIM drugs identified, MALPIP criteria identified a total of 476 PIMs, which was nearly threefold higher than the number of PIMs detected by the Beers (*n* = 151) and STOPP (*n* = 133) criteria.

The most common types of PIMs detected by the MALPIP list were the use of frusemide (*n* = 66), hydrochlorothiazide (*n* = 47), pantoprazole (*n* = 39), prazosin (*n* = 20), warfarin (*n* = 14), enoxaparin (*n* = 13), and dabigatran (*n* = 12) (Additional file 1: Table S4).

## Discussion

This study describes the first Malaysian explicit criteria for identifying PIM to guide deprescribing among older adults. MALPIP serves as a comprehensive list of medications that older people should avoid or consider using with caution due to the potential harms and aid in medication selection. 91 medications which were not registered in the Malaysian market were excluded to account for practicality and feasibility of implementing deprescribing recommendations in the local setting.

Our list includes 169 criteria, of which 26 had not been identified in previous PIM lists. While there was some degree of overlap between the four criteria and the MALPIP, we also identified some differences. For instance, monoclonal antibodies were not listed as independent PIM in the BEERS [9], STOPP–START [10], PRISCUS criteria [19] and French criteria [20]. Monoclonal antibodies have been reportedly associated with increased risk of severe infections and malignancies [24], suggesting the need to use with caution in older adults after judging risk and benefits. As such, the MALPIP criteria provides a timely and comprehensive update of the PIM relevant to both Western and Asian settings.

There are several strengths in our study. The current study systematically reviewed PIM criteria published in Asian countries in addition to the STOPP–START criteria [10], Beer’s criteria [9], PRISCUS criteria [19] and French criteria [20], offering generalizability in the global context. We also took into consideration the pharmacovigilance data from the National Pharmaceutical Regulatory Agency, which enables the identification of PIM based on real-world adverse events of medications used among older populations, which was previously overlooked by most studies [25]. In addition, this study proposed a list of omitted drugs that could be restarted, an element which was absent in most explicit PIM criteria [25]. The MALPIP reported good sensitivity in detecting PIM in a Malaysian population.

It is crucial to consider the limitations of this study when interpreting the findings. The initial draft of the PIM list was developed based on previously published criteria, which could limit its timeliness and completeness. Nonetheless, this was mitigated using the pharmacovigilance data to support the identification of potential PIM from newer generation drugs. We did not propose an explicit list of drug–drug interactions, as similar drug–drug interactions may also happen in younger population, and data are easily available in most drug–drug interactions checker. We also did not propose alternatives drugs for the listed PIM, as most of the alternatives could also be potentially inappropriate to older population.

For most criteria, consensus emerged during the Delphi, where in most cases drugs were either listed as a PIM/PPO, or not a PIM/PPO. A consensus-based decision was sought through group discussion for medications that were unequivocal. It is also worth noting that there are 11 medications that were undecided after the second Delphi round. To achieve consensus, we conducted an interview to obtain geriatricians’ opinion regarding the undecided PIM candidates, to enhance the accuracy and comprehensiveness of the MALPIP criteria. This is in line with the GRADE guidelines, which recommend the incorporation of a variety of considerations, including expert opinion, clinical judgement, and context in the decision [26].

Pharmacovigilance is important to recognize and monitor adverse drug reactions [27]. Given this important function, we systematically analyzed the adverse drug events experienced by older adults. Through this exercise, we identified 2 medication classes and 17 medications as PIM candidates. From these, we included hydroxychloroquine as PIM, due to the life-threatening adverse events and QT prolongation [28]. This underscores the need to perform baseline and periodic electrocardiograms to assess the patient's cardiac function before initiation and during follow-up, whenever indicated.

The MALPIP list can be useful as a supporting tool for doctors, pharmacists, and other healthcare providers to guide appropriate prescribing and deprescribing. Importantly, the MALPIP list should not be considered as a complete PIM list, and users must evaluate the risk and benefits tailored to the functional status, clinical profile, prognosis, and individual needs of the older patients. In some PIMs identified, such as antipsychotics, these medications could be indicated in patients with delirium and uncontrolled aggressive behaviours [29]. Likewise, the use of low-dose digoxin could be beneficial in heart failure [30]. The practice statements accompanying the PIM in the Tables aimed to inform clinicians regarding the potential risks and precaution to take if the medication is indicated.

Like most existing criteria, the MALPIP are designed to be used among older adults aged 60 years and above due to the higher risk of harms associated with this population. While it would have been ideal that a different cutoff be applied to different population, e.g., those who have multimorbidity and frailty versus robust older adults, evidence base for such criteria is often insufficient to set such a threshold [31, 32]. As such, it is important for clinicians to exercise sound judgment and apply these criteria in clinical settings with a practical approach.

The current MALPIP list required further validation, by examining its feasibility through identification of real-world PIM-related adverse events, reduction of adverse events and improvement of clinical outcomes.

## Conclusion

The MALPIP list serves as an evidence-based reference for prudent prescribing practices among older adults. Furthermore, it acts as an efficient tool to guide deprescribing, thereby minimizing inappropriate medications within this population. However, clinicians must exert risk and benefits evaluation based on individual patient profile before starting or stopping any medication. Future studies should consider validating the ability of the MALPIP list in identifying PIM, optimizing prescribing, and improving patient’s clinical outcome.

### Supplementary Information

**Additional file 1: Table S1.** List of 118 medications that were merged into 36 classes. **Table S2.** Independent PIM list and practice statements. **Table S3.** Disease-specific PIM list and practice statements. **Table S4.** Types of PIM identified in the validation data set.

## Data Availability

All data to reproduce the tables and figures in the manuscript and Additional Information can be obtained with reasonable request from the corresponding author.
