# Supplementary material for: Development of a Malaysian potentially inappropriate prescribing screening tool in older adults (MALPIP): a Delphi study
Source: J Pharm Policy Pract. 2023 Oct 19;16:122. doi: 10.1186/s40545-023-00630-4 (PMC10588247; doi:10.1186/s40545-023-00630-4)
Supplement: Supplementary file 1 — Additional file 1: Table S1. List of 118 medications that were merged into 36 classes. Table S2. Independent PIM list and practice statements. Table S3. Disease-specific PIM list and practice statements. Table S4. Types of PIM identified in the validation data set. [file 40545_2023_630_MOESM1_ESM.docx]

**Table S1: List of 118 medications that were merged into 36 classes**

|  | H2-receptor antagonists | Cimetidine, famotidine |
| --- | --- | --- |
|  | Proton pump inhibitors | Omeprazole, esomeprazole, rabeprazole, pantoprazole |
|  | Sulfonylureas | Glyburide (glibenclamide), glimepiride, glipizide, gliclazide, |
|  | Vitamin K antagonists | Warfarin |
|  | Direct thrombin inhibitors | Dabigatran |
|  | Factor Xa inhibitors | Rivaroxaban, apixaban |
|  | Antiarrhythmic | Amiodarone, flecainide |
|  | Loop diuretics | Furosemide, bumetanide |
|  | Thiazide diuretics | Hydrochlorothiazide, indapamide |
|  | Non-selective beta blockers | Propranolol, labetalol, sotalol |
|  | Non-dihydropyridine calcium channel blocker | Diltiazem, verapamil |
|  | Dihydropyridine calcium channel blocker | Short acting nifedipine |
|  | Oral oestrogens | Oestradiol, estriol, tibolone |
|  | Androgens | Testosterone |
|  | Phosphodiesterase-5 inhibitors | Sildenafil, tadalafil, vardenafil |
|  | Antimuscarinics for urinary frequency and incontinence | Oxybutynin, solifenacin, tolterodine |
|  | Selective alpha-1 blockers | Tamsulosin, alfuzosin, terazosin |
|  | Systemic corticosteroids | Methylprednisolone, prednisolone, betamethasone, dexamethasone, hydrocortisone |
|  | Aminoglycosides | Gentamicin, amikacin |
|  | Monoclonal antibodies | Rituximab, bevacizumab |
|  | Non-COX-2 selective NSAIDs | Benzydamine, diclofenac, ibuprofen, indomethacin, ketoprofen, ketorolac, mefenamic acid, meloxicam, naproxen, piroxicam |
|  | COX-2 selective inhibitors | Etoricoxib, celecoxib |
|  | Opioids | Meperidine (pethidine), pentazocine, morphine, tramadol, methadone |
|  | Barbiturates | Phenobarbitone |
|  | Dopamine agonists | Pramipexole |
|  | Barbiturates with hypnotic properties | Pentobarbital |
|  | Atypical antipsychotics | Clozapine, olanzapine, amisulpiride, risperidone, aripiprazole, paliperidone, quetiapine |
|  | Benzodiazepines | Alprazolam, bromazepam, clobazam, clonazepam, diazepam, lorazepam, midazolam, nitrazepam |
|  | Thioxanthones | Flupentixol, zuclopenthixol |
|  | First-generation antipsychotics | Chlorpromazine, prochlorperazine, fluphenazine, perphenazine, trifluoperazine, sulpiride |
|  | Selective serotonin reuptake inhibitors (SSRIs) | Paroxetine, fluoxetine, fluvoxamine, sertraline |
|  | Acetylcholinesterase inhibitors | Donepezil, rivastigmine, galantamine |
|  | Tricyclic antidepressants | Imipramine, clomipramine, amitriptyline, doxepin |
|  | Xanthine derivatives | Theophylline, aminophylline |
|  | Anti-muscarinic bronchodilators | Ipratropium, tiotropium |
|  | First-generation antihistamines | Brompheniramine, chlorpheniramine, dexchlorpheniramine, dimenhydrinate, diphenhydramine, hydroxyzine, ketotifen, promethazine, triprolidine |

**Table S2: Independent PIM list and practice statements**

|  | **PIM criteria** | **Practice statements** |
| --- | --- | --- |
|  | **Acid related disorders (A02)** |  |
|  | H2 receptors antagonists | Increase risk of altered mental status. Start with low dose and taper up when indicated |
|  | Proton pump inhibitors | Increase risk of osteoporotic-related fractures, Clostridium difficile infection, community-acquired pneumonia, kidney disease, and dementia. Avoid duration more than 8 weeks. |
|  | Metoclopramide | May cause extrapyramidal symptoms and cognitive impairment. |
|  | Atropine | Anticholinergics effect increase the risk of falls and cognitive impairments |
|  | Belladona alkaloids | Anticholinergics effect increase the risk of falls and cognitive impairments |
|  | Clidinium-chlordiazepoxide | May cause sedation, weakness, confusion and hypotension. Contraindicated in patient with angle-closure glaucoma. Interact strongly with CNS depressant and opioids, causing respiratory depression. |
|  | Dicyclomine | Anticholinergics effect increase the risk of falls and cognitive impairments |
|  | Homatropine | Anticholinergics effect increase the risk of falls and cognitive impairments. May cause confusion. |
|  | **Constipation (A06)** |  |
|  | Viscous paraffin | Because of the risk of aspiration, oral liquid paraffin should not be given to patients who have difficulty swallowing, or to those with impaired neurodevelopment. Avoid immediately before bed and avoid prolonged use |
|  | Cascara sagrada | Contraindicated in abdominal pain, nausea, vomiting, faecal impaction, bowel obstruction, and inflammatory conditions of the intestine |
|  | Magnesium oxide | Risk of electrolyte disturbance and dehydration. Use with caution in older people and in renal impairment or cardiovascular disease. |
|  | Polyethylene glycol | Acute exacerbation of chronic heart failure and metabolic acidosis |
|  | **Diabetes mellitus (A10)** |  |
|  | Sulfonylureas | Avoid long-acting sulfonylureas. Can cause severe hypoglycaemia. |
|  | Pioglitazone | Can cause osteoporosis and fractures; may worsen heart failure |
|  | Insulin sliding scale | Similar risk of hypoglycaemia and poorer glucose control |
|  | **Antithrombotic agents (B01)** |  |
|  | Dipyridamole | May cause orthostatic hypotension. Caution in patients with aortic stenosis, unstable angina or recent MI due to vasodilation effects |
|  | Vitamin K antagonists | High bleeding risk and may cause cognitive decline |
|  | Direct thrombin inhibitors | Increased risk of gastrointestinal bleeding; haemorrhagic stroke and haemorrhage. Avoid use in elderly patients with CrCl less than 30 mL/min |
|  | Factor Xa inhibitors | Increased risk of gastrointestinal bleeding. Increased serum concentrations may occur with renal impairment and dose adjustment may be indicated. Assess renal profile before initiation. |
|  | Ticlopidine | Increased risk of bleeding, leukopenia and hepatotoxicity |
|  | Prasugrel | Increased risk of bleeding in older patients. Use with caution in severe renal and hepatic impairment. |
|  | **Cardiac therapy (C01)** |  |
|  | Digoxin | Low dose digoxin may be indicated in heart failure. Evaluate renal function and electrolytes before initiation. Watch for signs of toxicity (e.g. anorexia, nausea or confusion); monitor concentration if renal impairment, hypoxia, hypothyroidism or electrolyte disturbance is present. |
|  | Antiarrhythmics | May increase the risk of bradycardia, orthostatic hypotension, urinary retention, and falls. Examine drug-drug interactions. Tailor regimen according to patient's physiology and medication profile. Routine blood test and ECG warranted. |
|  | **Antihypertensives (C02)** |  |
|  | Methyldopa | May cause hypotension and increase fall risk |
|  | Clonidine | May cause hypotension and increase fall risk |
|  | Moxonidine | May cause hypotension and increase fall risk |
|  | Doxazosin | May cause hypotension and increase fall risk |
|  | Prazosin | May cause hypotension and increase fall risk |
|  | Reserpine | May cause hypotension and increase fall risk |
|  | **Diuretics (C03)** |  |
|  | Loop diuretics | Increase risk of falls, urinary incontinence, acute kidney injury, electrolyte imbalances. Should not be used as first-line agent |
|  | Thiazide diuretics | May cause hyponatremia, hypokalaemia, hyperuricemia and hypercalcaemia. Baseline serum electrolyte measurement and routine monitoring of serum electrolytes warranted. |
|  | Spironolactone | Increase the risk of hyperkalaemia. Initiate with low dose and monitor serum level frequently, especially in impaired kidney function. |
|  | **Beta blocking agents (C07)** |  |
|  | Non-selective beta-blocker | May increase fall risk. Consider using selective beta-blockers if indicated. |
|  | **Calcium channel blockers (C08)** |  |
|  | Non- dihydropyridine calcium channel blocker | May increase the risk of bradycardia and interact with digoxin, beta-blockers |
|  | Dihydropyridine calcium channel blocker | Higher risk of cardiac and all-cause mortality |
|  | **Sex hormones and modulators of genital systems (G03)** |  |
|  | Oral oestrogens | Contraindicated in patients with hepatic impairment or disease. Dose, duration and type of oestrogen should be evaluated on case-by-case basis |
|  | Androgens | May cause polycythaemia and sleep apnoea |
|  | Megestrol | Risk of thromboembolic events |
|  | **Urological (G04)** |  |
|  | Phosphodiesterase-5 inhibitors | May cause hypotension. Contraindicated in nitrate users. Use with caution in patients on vasodilators and diuretics. |
|  | Antimuscarinics for urinary frequency and incontinence | Due to anticholinergic effects, may increase risk of falls and delirium |
|  | Selective alpha-1 blockers | Postural hypotension and dizziness may happen but less common than prazosin. Closely supervise those on antihypertensive treatment because of possible additive hypotensive effects. |
|  | **Pituitary and Hypothalamic Hormones and Analogues (H01)** |  |
|  | Desmopressin | May cause hyponatremia. Monitor electrolyte imbalances if initiated. |
|  | **Corticosteroids for systemic use (H02)** |  |
|  | Systemic corticosteroids | Can use for short-term if indicated. Multiple adverse events such as altered glucose level and impaired immunity if used for long-term. |
|  | **Antibacterial for systemic use (J01)** |  |
|  | Nitrofurantoin | Avoid use in long-term suppression of infections and in patients with a CrCl less than 30 mL/min due to potential for pulmonary toxicity, hepatoxicity, and peripheral neuropathy, use safer alternatives. |
|  | Vancomycin | Greater risk for nephrotoxicity, especially with concurrent aminoglycoside administration. Dose adjustment and monitoring recommended |
|  | Clindamycin | Elderly with severe illness may be less tolerant of diarrhoea; monitoring of bowel frequency and fluid support recommended |
|  | Aminoglycosides | Increase risk of nephrotoxicity |
|  | **Antineoplastic agents (L01)** |  |
|  | Growth hormone | May impair glucose control and cause soft tissue oedema, arthralgias, carpal tunnel syndrome, and gynecomastia |
|  | Monoclonal antibodies | May cause oedema, cardiac complications, haematologic side effects, hepatic enzyme derange, nephrotoxicity. |
|  | **Immunosuppressants (L04)** |  |
|  | Leflunomide | Raised liver enzymes, headache, increased risk of infection. Contraindicated in severe hepatic impairment. Routine blood test warranted |
|  | Methotrexate | Myelosuppression, pulmonary infiltrates, hepatic fibrosis, nephrotoxicity and cirrhosis. Routine blood test, dosage adjustment and baseline chest radiography warranted. |
|  | Azathioprine | Common gastrointestinal, dermatological and hematologic side effects. Dose adjustments warranted |
|  | Etanercept | May increase risk of infections and worsening heart failure. Baseline blood test warranted. |
|  | **Anti-inflammatory and antirheumatic products (M01)** |  |
|  | Non-COX-2 selective NSAIDs | Increase risk of gastrointestinal and renal side effects. Use at the lowest effective dose for the shortest period of time if indicated. |
|  | COX-2 selective inhibitors | Increase risk of gastrointestinal and cardiovascular side effects. Use at the lowest effective dose for the shortest period of time if indicated. |
|  | **Muscle relaxants (M03)** |  |
|  | Baclofen | Increase risk of injury and delirium |
|  | Orphenadrine | Due to anticholinergic effects, may cause drowsiness, confusion and falls |
|  | Chlorzoxazone | Due to anticholinergic effects, may cause drowsiness, confusion and falls |
|  | **Antigout preparations (M04)** |  |
|  | Colchicine | Diarrhoea is common. Use with caution and reduce dose in older adults with CrCl less than 30 mL/min due to increased risk of bone marrow toxicity, gastrointestinal and neuromuscular adverse events |
|  | **Analgesics (N02)** |  |
|  | Opioids | May increase fall risk due to sedation and cognitive impairment. Regular evaluations if initiated, start with a lower dosage, and use a safer alternative if possible |
|  | Ergotamine and derivatives | May cause ischemia, overuse headache, paraesthesia, and ergotism |
|  | Fentanyl patch | Not recommended for opioid-naïve patients due to respiratory depression |
|  | **Antiepileptics drugs (N03)** |  |
|  | Barbiturates | High potential for physical dependence and risk of overdose at low dosages. May develop tolerance. |
|  | Phenytoin | Risk of neurology and cardia toxicity |
|  | Lithium | May affect cognitive function, cause hypothyroidism, impair renal functions |
|  | Sodium valproate | Reduced drug clearance in elderly, may induce irritability and depression. Review medication profile for drugs interactions if initiated |
|  | **Antiparkinson agents (N04)** |  |
|  | Trihexyphenidyl (benzhexol) | May cause delirium and confusion. |
|  | Biperiden | Due to anticholinergic effects, may cause sedation and falls |
|  | Benztropine | May cause confusion, blurred vision and behavioural changes. Start at low dose if indicated. |
|  | Selegiline | Avoid in CrCl less than 30 mL/min and ESRD, severe hepatic impairment. Interact with pethidine, moclobemide, fluoxetine and other tricyclic antidepressants. |
|  | Levodopa or dopamine agonists | CNS side effects confusion, hallucinations, delusions, psychosis, and agitation. Contraindicated with MAOIs and interact with anticholinergics. Routine blood tests warranted |
|  | **Neuroleptics (N05)** |  |
|  | Barbiturates with hypnotic properties | High potential for physical dependence and risk of overdose at low dosages. May develop tolerance. |
|  | Atypical antipsychotics | Increase risk of death, metabolic effects, orthostatic hypotension and cardiac arrythmias |
|  | Thioxanthones | May increase fall risk and induce Parkinsonism |
|  | Chloral hydrate | May cause prolonged sedation, cardiac toxicity and hypotension |
|  | Benzodiazepines | Significantly increase the risk of falls. Prescribe as short-term therapy and use short-acting agent if indicated. |
|  | Phenothiazines (first generation antipsychotics) | May cause extrapyramidal symptoms, orthostatic hypotension, delirium, QT elongation and sedation. Monitoring of blood pressure and electrocardiogram warranted. |
|  | Haloperidol | Increase risk of extrapyramidal symptoms, sedation, falls, stroke and all-cause mortality. |
|  | Zopiclone | Risk of abuse, dependence and withdrawal |
|  | Zolpidem | Risk of abuse, dependence and withdrawal |
|  | Hydroxyzine | Sedative effects may increase the risk of falls |
|  | **Neuroanaleptics (N06)** |  |
|  | Selective serotonin re-uptake inhibitors (SSRI’s) | May increase the risk of falls, osteoporosis and fractures. Exert caution in switching and deprescribing antidepressants. |
|  | Acetylcholinesterase inhibitors | Avoid use in older adults with syncope as may increase the risk of or bradycardia. May aggravate weight loss, seizures, Parkinson’s disease, asthma or COPD. Interact with anticholinergics and QT prolongation drugs. Avoid in severe renal and hepatic impairment |
|  | Tricyclic antidepressants | Increase risk of falls and fractures |
|  | Piracetam | May cause anxiety, insomnia, agitation, depression, drowsiness, and weight gain. |
|  | Methylphenidate | My increase the risk of cardiovascular events |
|  | **Antivertigo (N07)** |  |
|  | Flunarizine | Contraindicated in people with a history of depression, may exacerbate Parkinson's disease or other extrapyramidal disorders. Dosages higher than 5 mg/day can lead to an increased risk of extrapyramidal symptoms. |
|  | Cinnarizine | Parkinsonism such as tremor and rigor are infrequently reported, especially when greater than 150 mg/day |
|  | **Antimalarials (P01)** |  |
|  | Hydroxychloroquine | May cause prolonged QT, retinal toxicity and macular degeneration. Significant drug interactions. Review medication profile before initiation. |
|  | **Drugs for obstructive airway diseases (R03)** |  |
|  | Xanthine derivatives | Higher risk of toxicity in older adults. Avoid use as first-line. |
|  | Anti-muscarinic bronchodilators | May exacerbate narrow-angle glaucoma, intraocular pressure and prostatic hyperplasia, especially when used long-term |
|  | **Antihistamines for systemic use (R06)** |  |
|  | First generation antihistamines | Sedative effects increase the risk of falls |

**Table S3: Disease-specific PIM list and practice statements**

| **System/Condition** | **Medications** | **Practice statements** |
| --- | --- | --- |
| **Circulatory system** |  |  |
| Heart failure | Non-COX-2 selective NSAIDs, COX-2 selective NSAIDs, thiazolidinediones | May exacerbate heart failure |
|  | Non dihydropyridine calcium channel blockers (diltiazem, verapamil) | Contraindicated in systolic dysfunction. |
|  | Digoxin | Low dose digoxin may reduce hospitalization due to heart failure. Check renal function and electrolytes before initiation. |
|  | Tricyclic antidepressants | Affect cardiac contractility, associated with worsening of ischemic heart disease and sudden cardiac death |
|  | Sildenafil, tadalafil, vardenafil | May cause hypotension. Contraindicated in nitrate users. |
| Syncope | Donepezil, rivastigmine, galantamine | Increase the risk of bradycardia and syncope. If necessary, gradual dose increment and pre-treatment ECG recommended. |
|  | Non-selective alpha-blockers | Increase the risk of syncope and hypotension. If necessary, give the first dose at bedtime. |
|  | Tricyclic antidepressants, benzodiazepines, antipsychotics | Increase risk of hypotension |
|  | Methyldopa | Increase risk of hypotension. Could be indicated in resistant hypertension. If necessary, consider low dose initiation at 250 mg and maximum daily dose of 1000 mg. |
|  | Venlafaxine | May cause orthostatic hypotension. Measure standing and sitting BP, and baseline ECG in existing cardiac disease. |
| Bradycardia | Beta-blockers | Start with low dose and titrate up to tolerable dosage. |
|  | Donepezil, rivastigmine, galantamine | Increase risk of hospitalization due to bradycardia. Monitor heart rate if started. |
|  | Non dihydropyridine calcium channel blockers (diltiazem, verapamil) | Increase the risk of bradycardia |
| Arrythmias | Antiarrhythmics | Increase the risk of bradycardia. Examine drug-drug interactions , routine blood test and ECG if started. |
|  | Tricyclic antidepressants | May cause arrhythmias |
|  | Antipsychotics | May cause arrhythmias |
|  | Donepezil | May cause arrhythmias, reversible torsade’s de pointes and acquired long Q-T syndrome |
|  | Quinine | May induce arrhythmia and prolong QT-interval |
| Hypertension | Loop diuretics | Increase risk of falls, urinary incontinence, acute kidney injury, electrolyte imbalances. |
|  | Non-selective alpha blockers | May cause hypotension and syncope. Should not be considered as first line agent. |
|  | NSAIDs | May increase blood pressure. Consider other alternatives if possible. Monitor blood pressure if initiated. |
|  | Thiazides | May cause electrolyte imbalances. Baseline serum electrolyte measurement and routine monitoring warranted. |
|  | Methyldopa | Increase risk of hypotension. Could be indicated in resistant hypertension. If necessary, consider low dose initiation at 250 mg and maximum daily dose of 1000 mg. |
| Hypertension and urinary incontinence | Loop diuretics | May exacerbate urinary frequencies and incontinence and may affect quality of life. Substitute with other types of diuretics. |
| Dependent ankle oedema | Loop diuretics | Increase risk of falls, urinary incontinence, acute kidney injury, electrolyte imbalances. |
|  | Calcium channel blockers | Increase risk of ankle oedema. Considering dosage adjustment, switching CCB or switch to other classes if necessary. |
| Hypokalemia | Thiazide | May cause electrolyte imbalances. Baseline serum electrolyte measurement and routine monitoring warranted. |
|  | Digoxin | Concurrent use of digoxin with diuretics may increase risk of hypokalaemia |
|  | Fludrocortisone | Hypokalaemia if used long-term (> 8 months) |
|  | Loop diuretics | Increase risk of hypokalaemia. Examine unindicated potassium use in patients whose loop diuretics were deprescribed. |
| Hyponatremia | Thiazides | Increase risk of electrolyte imbalances |
|  | Selective serotonin reuptake inhibitors | May cause hyponatremia |
| Hypercalcemia | Thiazides | Increase risk of electrolyte imbalances |
| Hyperkalemia | ACE inhibitors | May cause hyperkalaemia |
|  | ARBs | May cause hyperkalaemia |
|  | Spironolactone | Increase the risk of hyperkalaemia. If indicated, initiate with low dose and monitor serum level frequently, especially in impaired kidney function. |
| High bleeding risk (e.g. coagulopathy) | Antiplatelet, vitamin K antagonists, direct thrombin inhibitors, direct factor Xa inhibitors | Prescribe with caution and monitor bleeding risk if drug is indicated. Evidence-based bleeding management is warranted. |
|  | NSAIDs | Increase bleeding risk |
| First deep vein thrombosis (without continuing risks) | Vitamin K antagonists, direct thrombin inhibitors, direct factor Xa inhibitors | Anticoagulant remains the mainstay of therapy for DVT. Use with caution and consider deprescribing after 6 months. |
| Cardiac conduction abnormalities and heart block | Tricyclic antidepressants, digoxin, verapamil, amiodarone | May induce cardiac arrhythmias and heart block |
|  | Acetylcholinesterase inhibitors (e.g. donepezil, rivastigmine) | May cause and exacerbate heart block |
|  | Beta blockers | Contraindicated in second or third-degree atrioventricular block |
| Orthostatic hypotension | Non-selective alpha blockers, calcium channel blockers, long-acting nitrates, chlorpromazine, tricyclic antidepressants, hydralazine, thiazide, loop diuretics, dopamine agonists (except levodopa), selegiline, antipsychotics, SGLT-2 inhibitors | General: Increase risk of orthostatic hypotension  Antipsychotics: Dosage adjustment should be considered if initiated  SGLT-2 inhibitors: May cause volume depletion. To assess volume status and blood pressure before initiating treatment. |
| Acute coronary symptoms | COX-2 selective NSAIDs, Non-COX-2 selective NSAIDS | Increase risk of acute coronary events |
| Raynaud disease | Beta-blockers | May cause peripheral vasoconstriction |
| **Nervous system** |  |  |
| Delirium, dementia or cognitive impairment | Urological spasmolytic (e.g. oxybutynin, tolterodine), first-generation antihistamines, antiparkinson drugs, antiemetics, muscle relaxants (e.g. orphenadrine), tricyclic antidepressants, paroxetine, antipsychotics, disopyramide, intestinal antispasmodics (e.g. atropine, scopolamine), psychostimulants (methylphenidate), barbiturates, benzodiazepines, zolpidem, deferoxamine, testosterone, corticosteroids, dopamine agonist, antiepileptics | May cause delirium and worsen cognitive function. Deprescribe after considering risk-benefits ratio |
| History of falls or fractures | Antipsychotics, benzodiazepines, zolpidem, opioids, tricyclic antidepressants, antiepileptics (e.g. carbamazepine, phenytoin), first generation antihistamines, non-selective alpha blockers, non-selective beta-blockers, paroxetine, duloxetine, loop diuretics | Increase risk of falls due to sedative or hypotensive effects |
| Parkinson disease and Parkinsonism | Antipsychotics (except quetiapine, clozapine), antiemetics (e.g. prochlorperazine, promethazine, metoclopramide) | May worsen Parkinson symptoms |
|  | Benzhexol | Avoid use in prevention of antipsychotic-induced extrapyramidal symptoms and treatment of Parkinson disease, as more effective agents are available. |
| Behavioral and psychological symptoms in dementia | Antipsychotics | May use as last line. Start with lose dose and titrate up to minimum effective dose. Regular assessment warranted. Deprescribe gradually and monitor relapse. |
|  | Anticonvulsants | Risk outweighs benefit. Avoid routine use. |
|  | Benzodiazepines | May cause sedation and worsen cognition. Only for short-term use in acute crisis. Avoid using as hypnotics. |
| Sleep disorders or insomnia | Antipsychotics | Atypical antipsychotics could be useful in insomnia. However, avoid as first-line agent. |
|  | Psychostimulants (e.g. methylphenidate, caffeine), theophylline, pseudoephedrine | May cause insomnia |
|  | Benzodiazepines | Risk outweighs benefit |
|  | Barbiturates | High potential for physical dependence and risk of overdose at low dosages. May develop tolerance. |
|  | Tricyclic antidepressants | Associated with orthostatic hypotension, falls and cardiac side effects. |
|  | Z-hypnotics | Long-term safety not established |
| Benign essential tremor | Levodopa and dopamine agonists | Lack of efficacy evidence |
|  | Benzhexol | May cause delirium and confusion. |
| Depression | Centrally acting antihypertensives (e.g. methyldopa, reserpine) | May precipitate depression |
|  | Tricyclic antidepressant | Risk outweighs benefit |
| Seizures or epilepsy | Antipsychotics, bupropion, antidepressants, beta-lactam antibiotics, theophylline, H2 antagonists, levodopa, isoniazid, opioids | May induce seizure events |
| **Gastrointestinal system** |  |  |
| History of gastric or duodenal ulcers | Aspirin, Non-COX-2 Selective NSAIDs, Corticosteroids | General: If indicated, use with caution. Assess patient’s gastrointestinal risk factors and combine with PPI to reduce risk of ulcers |
| History of esophageal ulcers and dysphagia | Oral bisphosphonates | May cause oesophageal ulcers and esophagitis |
| Constipation | Drugs with anticholinergics properties, verapamil, oral iron, opioids, aluminium antacids, tricyclic antidepressants, calcium supplements | May cause constipation |
|  | Diuretics | May cause short-term constipation due to initial fluid loss |
| **Genitourinary system** |  |  |
| Chronic kidney disease | Non-COX-2 selective NSAIDs, COX-2 selective NSAIDs, glibenclamide, digoxin, dabigatran, metformin, oral bisphosphonates, colchicine, thiazide, factor Xa inhibitors, donepezil, memantine, cimetidine, ciprofloxacin, gabapentin, PPI, ACEI, ARBs, warfarin, allopurinol, opioids, aminoglycosides, amphotericin B, vancomycin, acyclovir, clopidogrel, ticlopidine | This list is not exclusive. Review the nephrotoxicity of other medications before initiation. |
| Benign prostatic hyperplasia | Strong anticholinergic drugs, tricyclic antidepressants, antimuscarinic bronchodilators (e.g. ipratropium, tiotropium) | Increase risk of urinary retention |
| Urinary incontinence | Non-selective alpha blockers, tricyclic antidepressants, diuretics | May exacerbate incontinence |
| Bronchial asthma | Non-selective beta blockers | May exacerbate asthma |
| **Respiratory system** | Benzodiazepine | May exacerbate asthma and increase mortality |
| Chronic obstructive pulmonary disease | Theophylline | Higher risk of toxicity in older adults. Do not use as first-line. Could be indicated in severe COPD. |
|  | Non-selective beta-blockers | Increase risk of severe exacerbations |
|  | Benzodiazepines | Increase risk of respiratory failure, suicidal ideation and mortality |
| Acute or chronic respiratory failures | Benzodiazepines, opioids, alcohol, cocaine, amphetamines | Small dose of opioids could be useful in palliative care. |
| Sleep apnea | Benzodiazepines | Increase the frequency and duration of apnoea |
| **Other systems** |  |  |
| Narrow angle glaucoma | Tricyclic antidepressants, antimuscarinic bronchodilators, strongly anticholinergic drugs, Urological spasmolytic (e.g. oxybutynin) | Increase intraocular pressure due to anticholinergic effects |
| Osteoarthritis and osteoporosis | Systemic corticosteroids (except intra-articular) | Short-term benefit in reducing pain and improve functioning. Multiple adverse events if used long-term, including altered glucose level and reduced immunity |
|  | High dose calcium supplements | Long term use of over 2 grams daily may cause toxicity such as hypercalcemia, renal calculi, hypophosphatemia, and nephrotoxicity, especially with individuals with chronic kidney disease. |
|  | NSAIDs | Assess patient's risk profile, routine evaluation of treatment response. Avoid long term use, use non-pharmacological or topical NSAIDs whenever possible. |
|  | Eperisone | Lack of efficacy evidence |
| Pain | Opioids in chronic mild pain, long-acting opioids without short-acting opioids in breakthrough pain | Use alternative analgesics in chronic mild pain. Short-acting opioids should be prescribed in breakthrough pain |
|  | Benzodiazepines | Significantly increased risk of emergency department visits |
|  | Tramadol | Increase risk of emergency department visits, falls/fractures, hospitalizations, and mortality |
| Gout | Loop diuretics | Increase risk of gout. Adequate hydration and routine uric acid level monitoring if initiated. |
|  | Colchicine | Diarrhoea is common. Use with caution and reduce dose in older adults with CrCl less than 30 mL/min due to increased risk of bone marrow toxicity, gastrointestinal and neuromuscular adverse events |
| Diabetes mellitus | Beta blockers | Increase risk of severe hypoglycaemia and cardiovascular events |
|  | Corticosteroids | Avoid >1 week without monitoring |
|  | Olanzapine, quetiapine, risperidone | Increase risk of diabetes |
| Breast cancer | Oestrogens | Increase risk of breast cancer relapse |
| Venous thromboembolism | Oestrogens | Increase risk of venous thromboembolism |

**Table S4: Types of PIM identified in the validation dataset**

| **Medications** | **Total** |
| --- | --- |
| Dexamethasone | 151 |
| Prednisolone | 78 |
| Frusemide | 66 |
| Hydrochlorothiazide | 47 |
| Pantoprazole | 39 |
| Prazosin | 20 |
| Warfarin | 14 |
| Enoxaparin | 13 |
| Dabigatran | 12 |
| Spironolactone | 9 |
| Terazosin | 9 |
| Omeprazole | 8 |
| Diphenhydramine | 7 |
| Tamsulosin | 7 |
| Tramadol | 7 |
| Ticlopidine | 7 |
| Rivaroxaban | 6 |
| Theophylline | 6 |
| Levodopa | 5 |
| Apixaban | 5 |
| Phenytoin | 5 |
| Diclofenac | 4 |
| Propranolol | 4 |
| Valproate | 4 |
| Colchicine | 4 |
| Doxazosin | 4 |
| Esomeprazole | 3 |
| Benzhexol | 3 |
| Diltiazem | 3 |
| Alfuzosin | 3 |
| Methotrexate | 3 |
| Leflunomide | 3 |
| Indapamide | 3 |
| Nifedipine | 3 |
| Amitriptyline | 3 |
| Methadone | 2 |
| Alprazolam | 2 |
| Risperidone | 2 |
| Amiodarone | 2 |
| Chlorpheniramine | 2 |
| Clonazepam | 2 |
| Verapamil | 2 |
| Digoxin | 1 |
| Dipyridamole | 1 |
| Methyldopa | 1 |
| Lorazepam | 1 |
| Fluvoxamine | 1 |
| Lorazepam | 1 |
| Donepezil | 1 |
| Ipratropium | 1 |
| Zolpidem | 1 |
| Haloperidol | 1 |
| Phenobarbitone | 1 |
| Aripiprazole | 1 |
| Quetiapine | 1 |
| Hydrocortisone | 1 |
| Tadalafil | 1 |
| Hydroxychloroquine | 1 |
| Total | 598 |
